# Residue Geometry Networks: A Rigidity-Based Approach to the Amino Acid Network and Evolutionary Rate Analysis

**DOI:** 10.1038/srep33213

**Published:** 2016-09-14

**Authors:** Alexander S. Fokas, Daniel J. Cole, Sebastian E. Ahnert, Alex W. Chin

**Affiliations:** 1 Theory of Condensed Matter Group, Cavendish Laboratory, 19 JJ Thomson Avenue, CB3 0HE, Cambridge, U.K

## Abstract

Amino acid networks (AANs) abstract the protein structure by recording the amino acid contacts and can provide insight into protein function. Herein, we describe a novel AAN construction technique that employs the rigidity analysis tool, FIRST, to build the AAN, which we refer to as the residue geometry network (RGN). We show that this new construction can be combined with network theory methods to include the effects of allowed conformal motions and local chemical environments. Importantly, this is done without costly molecular dynamics simulations required by other AAN-related methods, which allows us to analyse large proteins and/or data sets. We have calculated the centrality of the residues belonging to 795 proteins. The results display a strong, negative correlation between residue centrality and the evolutionary rate. Furthermore, among residues with high closeness, those with low degree were particularly strongly conserved. Random walk simulations using the RGN were also successful in identifying allosteric residues in proteins involved in GPCR signalling. The dynamic function of these residues largely remain hidden in the traditional distance-cutoff construction technique. Despite being constructed from only the crystal structure, the results in this paper suggests that the RGN can identify residues that fulfil a dynamical function.

Network analysis can increase our understanding of the behaviour of a system by applying mathematical algorithms to illuminate the patterns of interacting elements. Indeed, many areas of science are concerned with characterising how the components of a system interact and give rise to a behaviour or function. In particular, a central theme of structural biology is the intimate relationship between a protein’s structure and its function. Networks are therefore highly applicable to the study of proteins, as both of these aspects can be described in terms of networks. Furthermore, due to advances in computer science, there are now several approaches^1^ for characterising protein interactions and topology. We term these signatures “network-function relationships”, drawing inspiration from structure-function relationships often derived in biochemical experiments.

Early AANs were constructed using a physical distance-cutoff (DC)^2^, whereby edges are placed between residues that are within a certain DC. This method showed that AANs display small world properties, where few nodes are direct neighbours, but most nodes can be reached in few steps. The benefit of such small world properties, which is likely to be employed by proteins, is the ability to effectively distribute information. In addition, properties of such networks, including average degree and clustering coefficient, have been employed to score and subsequently discriminate between native and non-native structures^3^. While insightful, such AANs are considered coarse grained methods, as they only store information concerning the general protein shape. Therefore, although the DC construction technique requires low computational resources, it is at the expense of a failure to accurately model the chemical environment from which more advanced protein functions can potentially be inferred.

In recent models, the network-function relationship has evolved to account for motion, which is required to demonstrate functions such as allostery^4^, recognition^5^, and catalysis^6^. Protein motion ultimately relies on the strengths of chemical interactions within the environment. For an AAN to successfully provide insight into dynamical functions such as allostery, representation of the environment has to be extended beyond that of the DC method in an attempt to elucidate the cumulative effect of side-chain dynamics. The most commonly used dynamical techniques derive edge information from molecular dynamics (MD) simulations in which edges are introduced based on the percentage of conformations in which two residues are in contact during a simulation^7^,^8^. Employing molecular dynamics simulations certainly provides information on the chemistry of the environment, but at an added computational expense. A computationally cheap approach that uses only the static structure and maintains a comparable level of validity would be profitable for certain applications, in particular, for the *de novo* design of protein function.

In this paper, the parameters of the residue-residue interaction network are built by identifying, in the static crystal structure, the strongest non-bonded interactions. To do so, these interactions are analysed using the FIRST (Floppy Inclusions and Rigid Substructure Topography) rigidity analysis software^9^,^10^, which identifies hydrophobic tethers and quantifies the strengths of hydrogen bonds and salt bridges using a geometry-based scoring scheme. The information stored in the scoring function is used to (a) implement an energy cutoff such that only the strongest hydrophilic interactions, namely hydrogen bonds and salt bridges, are involved in the network and (b) construct weighted and unweighted networks. These networks allowed us to identify influential nodes by applying graph theoretical algorithms.

A similar approach to network construction has been employed previously using the computational tool BONGO^11^, which predicts the structural effect of single amino acid polymorphism. In their approach, nodes are removed iteratively to identify those that participate more strongly in building up the edges of a graph. By comparing this graph theoretical measure for wild-type and mutant proteins, BONGO is able to identify disease-associated mutations. Impressively, this algorithm is able to distinguish between disease-associated and non-disease-associated mutations with a positive predictive value and negative predictive value of 78.5% and 34.5%, respectively. Here, we build upon this approach by using the FIRST algorithm^12^ to estimate the energy of hydrogen bonds and salt bridges, thus allowing weighted networks to be built and facilitating the removal of less influential hydrogen bonds.

FIRST-generated interaction networks have been used in previous studies as input for constrained dynamical simulations. Critically, these dynamics were shown to agree with experimental protein motions^10^,^13^ and provided insight into a wide range of protein functions^14^,^15^. In particular, FIRST constraint-based dynamics of the HIV-1 trans-activation-responsive region RNA-bound structure were found to agree strongly with the fluctuations found in MD and NMR studies^16^. These constrained dynamic studies have also been used to investigate the flexibility of the nicotinic acetylcholine receptor ion channels, and led to the identification of key residues that are predicted to facilitate rapid communication between the binding site and the transmembrane gate^17^. Low-frequency motions in the constrained dynamics of a photosynthetic pigment-protein complex have also been studied and used to explain how conformal motion may promote efficient exciton energy transfer^18^. This suggests that a diverse range of functional dynamics can be readily simulated using this constraint-based technique, which uses as input the set of interactions that are employed to construct the network in the present study. Thus, in this paper, we propose a “pseudo-dynamical” construction of the amino acid network that uses the above geometrical analysis of the static structure. Similar to the use of FIRST-generated interactions as constraints in geometric simulation, we hypothesise that, to a good approximation, the supported dynamics of the protein structure is *encoded* in the interactions identified in its native state. If correct, this would allow dynamic functions to be investigated using the static structure. We call the AAN that results from this construction method a *residue geometry network* (RGN). In this paper, we have validated the ability of the RGN to predict functionally important residues based on comparison with the evolutionary rate, which we additionally use as a data set for optimising the weights.

Evolutionary rate (*dN/dS*) is calculated as the ratio between non-synonymous mutations in protein coding genes (*dN*), which change the amino acid sequence and are a function of the selective pressures, and synonymous mutations (*dS*), which do not affect the amino acid sequence and therefore remain neutral with respect to selection pressure. Using a previously assembled comprehensive data set^19^ we have investigated whether residue centrality is a major constraint on residue evolutionary rate. For the unweighted RGN (unRGN), where all network edges are assigned equal weights, we identified a strong, negative correlation between degree, betweenness, and closeness centrality measures and the evolutionary rate. Using the weighted RGN (wRGN), we find the same trend, as well as an increase in the weighted betweenness centrality correlation when compared to the correlation measured using the unRGN. We demonstrate the importance of added chemical insight using more complex network analytics to study dynamical functions. For example, residues that form few local connections while maintaining high global centrality are, unexpectedly, found to be more highly conserved than hub residues. The subtle dynamical role played by these residues, whose corresponding nodes form hinges in the RGN, is investigated using several proteins from the data set. To develop the theme of deriving dynamical functions from static structure, we have employed the expected visiting time (EVT), which measures node signal traffic during random walks through the network, to investigate the allosteric response in proteins involved in GPCR signalling. This method has previously been used in combination with molecular dynamics simulations to identify residues that regulate allostery^20^. Despite only using the crystal structure, residues that score high EVT in the RGN are found to overlap strongly with well-known regulators of the allosteric response. These residues are often not identified when applying EVT to the traditional distance-cutoff AANs. We hope that the ability to identify functional signatures in the RGN will broadly empower the scientific community with a low-cost approach to understand, modify, and design protein structure, and by association, function.

## Methods

### Measuring the Evolutionary Rate of High and Low Centrality Bins

The data set^19^ we have used to investigate the relationship between centrality and evolutionary rate consists of 795 proteins derived from structural homology mapping of yeast (*Saccharomyces cerevisiae*). In particular, the multiple sequence alignments were calculated using ClustalW to generate an alignment between a translated open reading frame (ORF) from *Saccharomyces cerevisiae*, the mapped protein structure subunit sequence, and orthologous ORFs from *Saccharomyces paradoxus, Saccharomyces mikatae*, and *Saccharomyces bayanus*. We have then employed the PAML software package^21^ to calculate the number of amino acid substitutions (*dN*) and the number of silent substitutions (*dS*). The latter therefore acts as a normalising factor. The ratio of these two factors, known as the evolutionary rate (*dN/dS*), gives insight into the rate of selection normalised by mutations at the DNA level. As the 4 species are closely related, a single value of the evolutionary rate was calculated for the entire tree. In particular, the module *codeml* within PAML was employed to calculate *dN/dS* using the tree [S. cerevisiae, S. paradoxus], S. mikatae, S. bayanus. *dN/dS* displays a greater validity for a larger data set^22^, and *dN/dS* for individual residues does not provide realistic insight. To improve the accuracy of our measurements, residues were sorted into bins to increase the number of codons being analysed and provide a suitably large signal, as has been done in previous experiments^19^.

At the most basic level, a network is a collection of nodes with links (also termed ‘edges’) representing interactions between them. We have written the script for building and analysing the RGN in the Python coding language^23^. Network analysis was achieved using the python package NetworkX^24^. For each protein, the corresponding network consisted of nodes, each representing one residue, and edges that corresponded to a particular interaction. When constructing the RGN, covalent interactions were represented by an edge between adjacent amino acids. The geometric tool FIRST (version 6.2.1, http://flexweb.asu.edu)^9^,^10^ was used to generate the non-covalent edge components of the network. FIRST identifies constraints dependent on bond lengths and angles, hydrophobic interactions, salt bridges, and hydrogen bonds. Proteins were downloaded from the protein data bank (http://www.rcsb.org) and a single chain was chosen for the analysis. Hydrogen atoms were added to the 795 proteins using the Reduce module within Molprobity^25^. Reduce does add hydrogens to ligands, but does not add explicit H atoms to water molecules. In the cases where H atoms of water molecules were not already present in the PDB file when downloaded, the water oxygen atoms were removed. The data set contains both X-ray crystallographic and NMR data. In the case of NMR structural ensembles, the ‘best conformer’, as identified in the PDB file, was used. If a specific conformer of the NMR ensemble was not specified in the PDB file, the first conformer was selected.

The default settings of FIRST (syntax -non) were used during the analysis of all protein structures. FIRST places hydrophobic “tethers”, for which the energy is not calculated, between aromatic or aliphatic sidechain carbon atoms that are within 4 Å of each other. The energies of hydrogen bonds are calculated using the donor-hydrogen-acceptor geometry. Salt bridge energy calculations employ a different energy function that reflects their greater bond strength and reduced directionality. Using the FIRST energy function, the strongest hydrophilic interactions have measured energies between −5 *kcal*/*mol* and −10 *kcal*/*mol*. Interactions with metals and other ions are treated as covalent bonds. Finer details of the treatment of the protein structures have been discussed elsewhere^10^. The hydrogen bond energy cutoff (H_*cut*_) controls the strength of the non-covalent interactions (excluding hydrophobic ones) involved in network construction. As hydrophobic interaction energies are not explicitly calculated by FIRST, varying H_*cut*_ does not remove any of the so-called hydrophobic tethers from the simulations. For example, if the H_*cut*_ = −2.0 *kcal*/*mol*, only hydrogen bonds and salt bridges with energies lower than −2.0 *kcal*/*mol* would be included in the RGN, in addition to all of the hydrophobic and covalent edges.

We have also constructed the amino acid network for the proteins in the data set using the DC technique. Using the Bio3D suite^26^, edges are placed between a C*α* atom and other C*α* atoms that lie within an imposed DC (in Å). The closeness centrality properties of this unweighted network were then investigated in an identical manner to the RGN.

**Figure 1:**
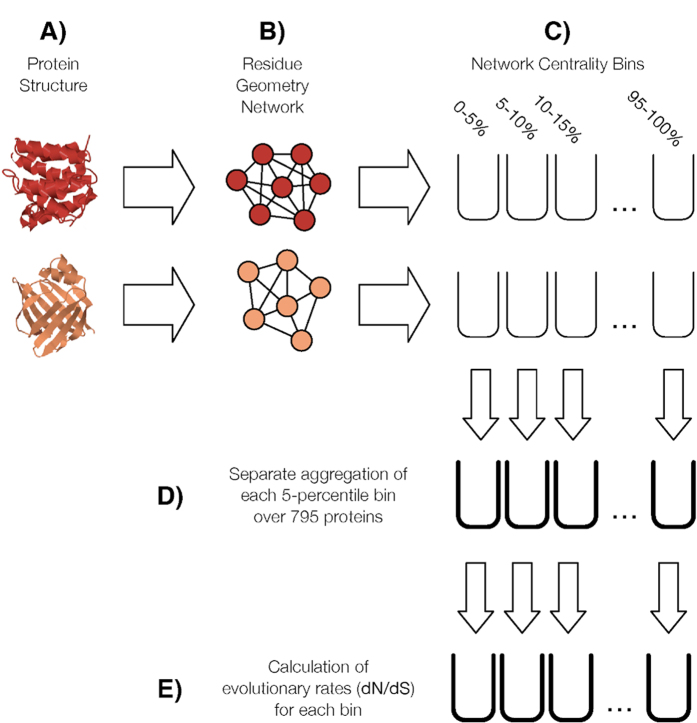
Evolutionary analysis method. For each protein, the centrality for all residues is calculated and assigned to one of 20 bins depending on the centrality within each protein. Equal 5-percentile bins are then aggregated, allowing an accurate measure for the evolutionary rate to be calculated for each of the 20 summed bins. An identical procedure has been previously employed^19^ using this data set, whereby a strong signal is attained by binning residues according to their relative solvent exposure, and the *dN/dS* is then calculated to look at “bin evolution”.

As FIRST uses an all-atom representation when identifying interactions, each node represents the interactions made by the atoms forming a particular residue in the polypeptide chain. The centrality of every node, and thereby each residue position in the dataset, was calculated, providing a dataset of 264,773 residue positions. Within *each* protein, the centrality of all the residues was calculated, allowing all residues to be ordered and partitioned into 20 bins according to their centrality value. Each of the bins represents 5% of the centrality values within a given protein; residues with the top 5% centrality values are assigned to bin 100, the highest 5–10% are assigned to bin 95, and so on, resulting in 20 ‘rank’ bins for each of the 795 proteins. The evolutionary rate within each bin (summed over the data set) was then calculated to study relationships between centrality and evolutionary rates (Fig. 1).

Centrality is an important concept in network analytics. Centrality attempts to identify nodes that are the most important, or influential, in a system. The centrality of a particular node can be measured using several different algorithms that highlight different aspects of the network. Degree centrality is simply the number of edges connected to a node. The assumption here is that a better connected node will be more important. However, this measure of centrality does not consider the position of the node relative to other nodes in the network. Betweenness centrality and closeness centrality are more complex measurements that account for the topology of the network. Betweenness centrality measures the involvement of a node in the shortest paths between all other pairs of nodes in the network. This measure is, generally, useful for identifying nodes that play a role in the flow of information. This property was calculated according to the equation:


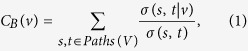


where *V* is the set of all nodes, *σ*(*s, t*) is the number of such shortest paths between nodes *s* and *t*, and *σ*(*s, t* | *v*) is the number of such paths that involve node *v*. Weighted and unweighted betweenness centrality are computed equivalently, with path lengths derived from weighted and unweighted networks, respectively. Weighted path length is computed according to:


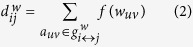


where *f* is a map from weight to length, *a* is the connection status of *u* and *v*, and 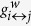 is the shortest weighted path between *i* and *j*. Closeness centrality is the inverse of the average shortest path length between residue *i* and all other residues in the network, according to the equation:


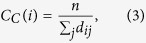


where *n* is the number of nodes in the system and *d*_*ij*_ is the shortest path length between nodes *i* and *j*.

**Figure 2:**
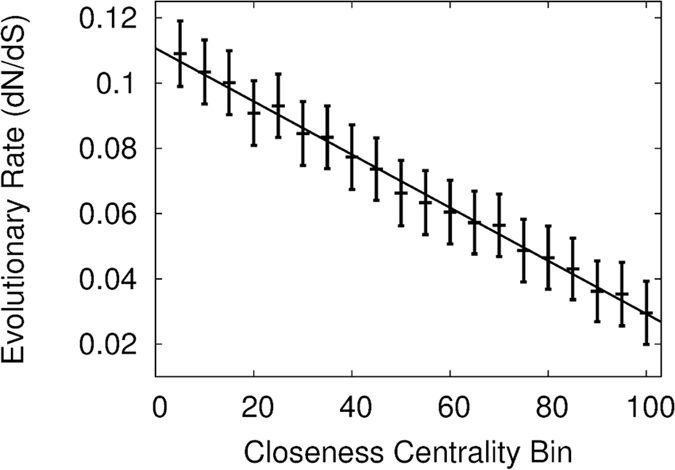
The correlation coefficient between the evolutionary rate and the centrality bin is used to assess whether the different forms of centrality influence the evolutionary rate of the residues in the data set. The Pearson correlation coefficient is −0.997 using an unRGN network with H_*cut*_ = −3 *kcal*/*mol* between the bin evolutionary rate and the closeness centrality bin. The trend line for the data is shown in black, with standard error bars displayed for each calculation.

The correlation coefficient was calculated between the bin evolutionary rate and bin number (Fig. 2). We note that if residues are randomly assigned to the ranked bins a correlation coefficient of 0.07 is found. To ensure validity of the results, the correlation coefficient of this plot was calculated while manually varying certain parameters (*e.g.* H_*cut*_). This allowed identification of the values that resulted in the best performance (strongest correlation) of the construction methods.

To construct the wRGN for analysis using the weighted betweenness centrality metric, covalent and hydrophobic network weights were identified that optimised the correlation with the evolutionary data. We varied covalent energies between 0 and −40 *kcal*/*mol* and hydrophobic energies between 0 and −4 *kcal*/*mol*. Increasing the search range beyond these limits would not improve the agreement with evolutionary rate. We found that the optimum weights were −2.5, −2.5 and −1 kcal/mol for hydrophilic, hydrophobic and covalent interactions respectively, as discussed on page 6.

### Measuring the EVT of the RGN

The expected visiting time (EVT) measures the importance of each residue in the transfer of information through the network^20^. Signals are initiated at a particular residue and undergo random walks with the likelihood of propagation between two nodes determined by the weight of the edge that connects them, before being absorbed at a second site. The EVT value for a residue is then calculated as the average visiting frequency of signals that pass through the corresponding node in the network for all absorbing sites. EVT analysis is therefore capable of identifying communication between distant sites and multiple pathways exploration, and has been previously used to study allosteric communication in proteins^20^.

A Markov transition matrix, **T**, was derived from the wRGN and used to determine the signal transition probability to the nodes interacting with the signal node. The transition probability from node *i* to *j* (T_*ij*_) is given a weight equal to the absolute value of the interaction between *i* and *j (α*_*ij*_) divided by the degree:


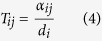


For hydrophilic interactions, the weight is equal to the interaction energy computed using FIRST. For hydrophobic and covalent interactions, weights that resulted in the strongest correlation of weighted betweenness centrality with evolutionary rate were employed (2.5 and 1.0 for hydrophobic and covalent weights, respectively).

The information flow through the wRGN is modelled using the absorbing Markov chain model^27^, where the *n* x *n* “fundamental matrix” of the corresponding absorbing Markov chain is calculated according to:


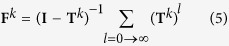


In the above, **T**^*k*^ is the reduced transition matrix after the kth row and column were removed. The EVT for all nodes is calculated by averaging **F**^*k*^ over all absorbing nodes k:


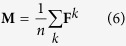


EVT values have a bias towards residues that are nearby, whereas allostery occurs between distant sites in the protein. Here, we scale the EVT values by multiplying the raw EVT value for each node by the shortest-path distance between the signal initiating site and the residue of interest^20^. The EVT values have been normalised to have a mean of 0 and standard deviation of 1.

## Discussion and Results

### Evolutionary Rate correlates with Residue Centrality

We have conducted the current study to measure the evolutionary rate of high and low centrality residues. Our AAN is constructed using FIRST-generated non-covalent interactions. The construction and analysis retains the inexpensive computational resources of widely used DC techniques while providing additional insight into the geometry and chemistry of the residue environment. In this work, we have investigated the relationship between high centrality residues, which have structural or functional significance^28^, and evolutionary rate.

Initially, we constructed the amino acid interaction network using the widely employed DC method. In particular, we have calculated the AAN for the protein data set using a DC of 4, 6, 8, and 10 Å. When constructing the AAN using a DC of 6 Å, an edge is placed between a residue and all residues that lie within 6 Å (measured between C*α* atoms). This is a commonly used technique for the analysis of protein structure^1^. In a previous study^19^, which employed the same data set, residue buriedness from the solvent was calculated and correlated with the evolutionary rate. A correlation coefficient of −0.996 was identified between buriedness and evolutionary rate. In our analysis, we found that closeness centrality bins grouped using a DC of 6 Å also resulted in a correlation coefficient of −0.996 between the closeness centrality bins and evolutionary rate. Of course, these two physical observables are related, as residues in dense regions of the protein are likely to be shielded from the solvent. While residue density (as calculated by DC) and buriedness from the solvent, are strong selective pressures, they do not account explicitly for residue-residue interaction strengths. Therefore, such techniques cannot provide an understanding of the interaction network that stems from the chemistry of the environment. We have turned to the RGN to design a static AAN construction technique that reflects these important aspects of the system.

We varied certain parameters of the simulation to investigate the effect on the accuracy of the RGN using the correlation coefficient between centrality bin and evolutionary rate as a meter, where a stronger correlation signified better performance of the RGN. In the unRGN, non-covalent and covalent interactions were all given the same weight and so the number of interactions is the only quantity that can be varied. The correlation between centrality and evolutionary rate was therefore optimised as a function of the H_*cut*_ (Fig. S1A), which controls the minimum energy of a hydrogen bond or salt bridge that will be involved in the analysis. The unRGN is a coarse approximation; the interactions that occur between protein residues in nature vary strongly, which is not reflected in the analysis as all covalent and non-covalent interactions are assigned the same weight. Nonetheless, a strong, negative correlation can be found between centrality bin and evolutionary rate. The optimum value of the H_*cut*_ for the unRGNs (degree, betweenness, closeness) can be found in Table 1. We observe similar correlation coefficients as was observed between residue buriedness and evolutionary rate^19^.

### Residue Composition of Centrality Bins

**Figure 3:**
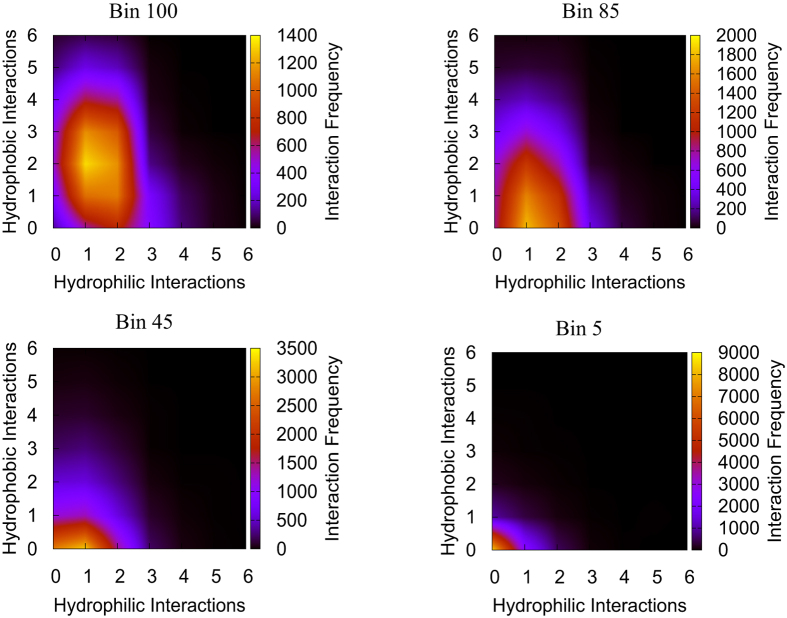
The number of hydrophilic and hydrophobic interactions made by residues in each bin (grouped according to unRGN closeness centrality) were investigated, with the results of bins 5, 45, 85, 100 portrayed here to represent the trend. While the number of hydrophilic interactions formed by the residues does not vary greatly until very low centralities, the number of hydrophobic interactions can be seen to steadily decrease as centrality decreases.

The residues belonging to the unRGN closeness centrality bins were analysed further to study the nature of the non-covalent interactions in the data set, as well as examine the aspects of protein structure that are evident from the RGN. It is interesting to note that the algorithm performs well when the number of hydrophilic interactions is similar to the number of hydrophobic ones (Fig. S1B). We expect this to be a general property of the data set as the number of hydrophilic and hydrophobic interactions strongly correlates with the size of the protein (Fig. S1C). Thus, balancing the influence of hydrophobic and hydrophilic interactions is achieved with a mid-range cutoff. The sigmoidal shape of the percentage of total interactions in each bin suggests that high centrality residues do exhibit a disproportionate number of interactions (Fig. S1D). Similar trends were also observed for degree and betweenness centrality bins. We expect this from the negative correlation between degree centrality and evolutionary rate, which suggests that residues forming few interactions have relaxed selection pressures. Indeed, the importance of ‘hub’ residues, which has been noted in previous studies^1^, is evident from the negative correlation between the degree centrality bins and evolutionary rate. This is due to the fact that higher centrality residues are often embedded deep in the protein (as opposed to the periphery), which is also evidenced by the DC analysis. We note that this is in line with the slower evolutionary rate of core residues relative to surface residues^19^. Furthermore, we find that residues displaying several hydrophobic and hydrophilic interactions have the lowest evolutionary rate (Fig. 3). This is likely to result from the smaller mutation space available for such residues that can form multiple hydrophilic and hydrophobic interactions.

Analysis of the strength of hydrophilic reactions in each bin (Fig. S2) revealed a steady increase in the average strength of the interactions despite not using weights in the analysis. However, residues with the highest closeness centrality bin have the highest standard deviation both for the average strength of an interaction, and for the number of interactions formed by each residue. This suggests not only that these residues generally employ stronger non-covalent interactions, but also that residues that have low degree centrality and/or form weak interactions are also found in the highest centrality bin.

**Figure 4:**
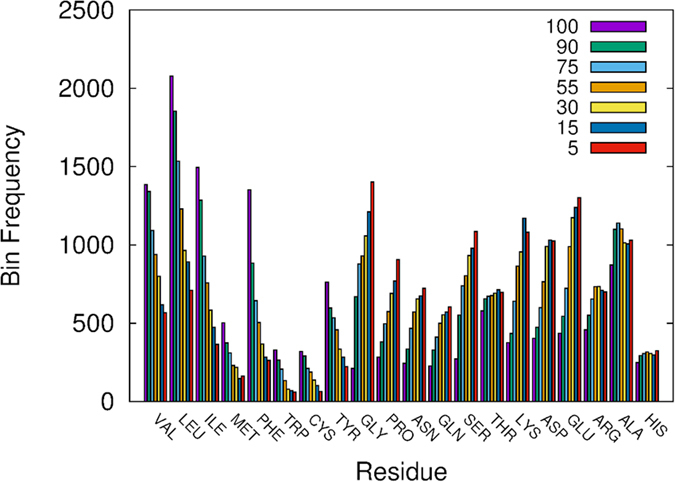
The frequency of residues per bin has been displayed in the above histogram. Clear trends can be seen for the majority of residues, either rising or falling, across the range of bins. In general, the frequency falls for residues that are larger and hydrophobic, and rises for residues that are smaller and polar. For example, the average molecular weight found for residues where the frequency falls is about 150, while for residues where it rises it is roughly 130.

The trends observed for the frequency of residue types in each bin (Fig. 4) agree with the distribution of residues in protein structures. For the majority of residues, the frequency is found to rise or fall with decreasing centrality. For hydrophobic residues, including Val, Leu, Ile, Tyr, Cys, Met, Trp, and Phe the frequency falls as centrality lowers while for Pro and Gly the frequency rises. For hydrophilic residues, namely Gln, Asn, Ser, Thr, we find that the frequency rises. The frequency also rises for all charged residues, namely Arg, Lys, Asp, Glu. In the case where the frequency falls with decreasing centrality it suggests that the residue, e.g. Leu, is more likely to be found in a high centrality region with a low evolutionary rate. Indeed, for hydrophobic residues, the observed trend reflects their central position in the protein. Similarly, charged residues are more likely to be found in low centrality regions with high evolutionary rate, which is in line with these residues being found near the surface of the protein. This illustrates the ability of the RGN to identify trends in protein architectures.

The discussed trends could be useful for identifying idiosyncratic residues in the RGN, namely those whose behaviour does not conform to the expected trends. For example, Gly is generally found in lower centrality bins, and therefore has a higher evolutionary rate. This suggests that, in the cases where the residue is found to have a high centrality, it plays an important role. This concept will be explored later using residues that have both a low local connectivity (low degree) yet maintain a strong global connectivity (high closeness). Taken together, the RGN is able to identify key trends in protein structure.

### Constructing the wRGN using weighted betweenness centrality

In addition to the unweighted networks, the wRGN was investigated for betweenness centrality. To do so, weights for the hydrophobic and covalent edges, which cannot be calculated within FIRST, were identified by optimising the correlation between weighted betweenness centrality and the evolutionary data. This required varying the hydrophobic interaction weight and the covalent bond weight parameters in addition to the H_*cut*_. In particular, all hydrophobic interaction weights and covalent bond weights were assigned the same value in each calculation, and were investigated in the range of 0 to −4 *kcal*/*mol* and 0 to −40 *kcal*/*mol*, respectively. After varying these parameters, we found the highest correlation with evolutionary rate (*r* = −0.997) with the following variables; H_*cut*_ = −2.5 *kcal*/*mol*, hydrophobic interactions = −2.5 *kcal*/*mol*, and covalent interactions = −1 *kcal*/*mol*, which correspond to edge weights of 2.5, 2.5, and 1, respectively. This is an improvement on the highest correlation between unRGN betweenness centrality and evolutionary rate (−0.995) (Table 1). We note that while the H_*cut*_ and the hydrophobic interaction strengths lie within the expected range, the covalent interaction energy is much lower than that found in nature. RGN edges are therefore not a straightforward representation of the enthalpic interactions that occur between the residues.

When constructing the network a question naturally emerges; what do the edges between residues represent? It is important to note that what we are modelling is not a static structure, but one that undergoes complex motions that have strong implications for function. While the backbone of covalent interactions helps determine the topology of the system, it is the non-covalent interactions that determine the unique ensemble of structures by restricting the conformational space accessible to the chain. Thus, we predict that the better performance of the diminished covalent bond weight (=1), compared to the order of magnitude higher strengths observed in nature, in the wRGN is simply a reflection of the low dynamic role such interactions have in comparison to non-covalent interactions with regards to the protein structure, where covalent interactions are relatively constant compared to the continual breaking and reforming of non-covalent interactions.

The makeup of the centrality bins in the RGN illustrates the additional information that can be gained with knowledge of the specific interactions found in the protein. As discussed, standard DC-construction techniques do not account for residue types and their specific chemical interactions. We will now discuss further attributes of the RGN, as evidenced by the evolutionary analysis and the wRGN network, that lie hidden when considering only DC AANs.

### Hinge Residues

We envisage residues with both a high closeness centrality and low degree centrality as “hinges” in the network. We have employed Constrained Network Analysis (CNA), which uses the FIRST rigidity tool to measure so-called rigidity indices, to investigate the flexibility of these residues. Rigidity indices are local calculations of stability that monitor at what energy (*kcal*/*mol*) a residue separates from a rigid cluster and becomes flexible^29^. Such transitions are related to the thermostability of proteins^30^. The low number of interactions made by RGN hinge residues suggests that, despite being well connected *globally* (as determined by high closeness centrality), these nodes support a greater amount of flexibility *locally* (low degree centrality). Indeed, we find a strong negative correlation between the degree centrality bin and rigidity index (Fig. S3).

**Figure 5:**
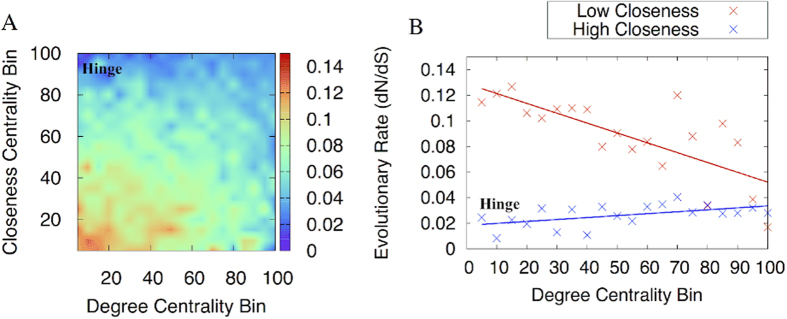
(**A**) Heat map displaying the evolutionary rate for degree - closeness centrality bins. (**B**) the high closeness centrality and low closeness centrality rows have been displayed with trend lines. The trend lines show clearly that as degree decreases, the evolutionary rate of residues with high closeness centrality decreases (*r* = 0.5, P value of 0.02) and the inverse trend is observed for residues with low closeness centrality (*r* = −0.7, P value of 0.0006). For the latter, when residues are randomly assigned to the ranked bins a correlation coefficient of −0.04 is found.

We decided to further explore the network hinges by binning residues according to closeness and degree centrality, resulting in 400 degree-closeness centrality bins (Fig. 5A). The correlation coefficient for the top (high closeness with increasing degree) and bottom (low closeness with increasing degree) row of Fig. 5A has been measured to show how evolutionary rate changes with decreasing degree (Fig. 5B). We can see that hinge residues are more highly conserved than residues that display both high closeness centrality and high degree centrality. This trend opposes what would be expected for decreasing degree alone, given the negative correlation between degree centrality bins and evolutionary rate. Indeed, the analysis of this trend using low closeness centrality bins paints a different picture. We have selected several residues to exemplify this observation.

**Figure 6:**
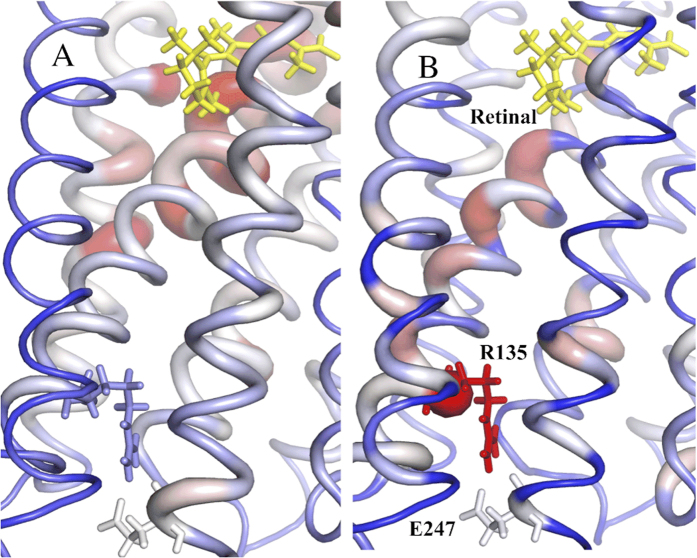
A portion of the RGN of GGPPS is shown, displaying interactions made by coloured nodes. It can be seen that G157 interacts with only two residues (low degree). However, these residues form extensive interactions with the environment, and therefore give rise to the high closeness of G157. Critically, the blue nodes are known to play important roles in ligand binding.

**Figure 7:**
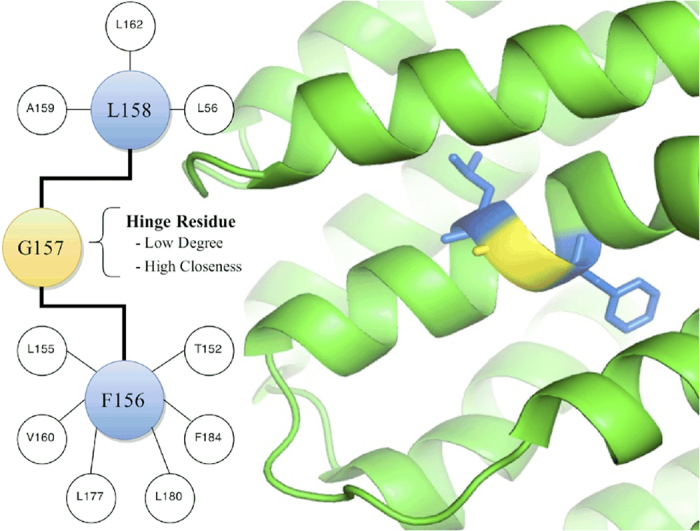
The DC-construction technique is unable to identify G157 as a residue with dynamical function as connections are formed between this residue and all residues within 6 Å. The distance labels in the above diagram show that the DC-construction technique results in an additional 6 edges to those observed in the RGN.

A well-conserved kinked *α*-helix is present in all geranyl-geranyl diphosphate synthase (GGPPS) protein structures^31^. This kink is found to occur just after residue G157 (Fig. 6) in a region of the protein that participates in ligand binding. G157 is found in the RGN analysis to form a hinge in the network: of 285 residues, it is ranked 7th highest for closeness centrality (0.182) and 270th for degree centrality (0.007). If we inspect the network more closely, we find two hub residues, namely F156 and L158, either side. F156 not only forms part of the ligand binding site^32^, but also coordinates several residues of the ligand binding site. The latter is also true for L158. G157 also lies within 3 degrees of two residues that bind the phosphate moiety of the ligand. The low degree centrality in the RGN suggests that this residue has high flexibility. Indeed, a low CNA rigidity index (−1.3 *kcal*/*mol*) was identified for G157. We speculate that, due to the close proximity to the active site and the binding pocket, this residue is involved in dynamics associated with ligand binding. Indeed, glycine flexibility has been proposed as a mechanism to support induced-fit structural movements during ligand binding^33^,^34^,^35^,^36^. This residue is not highlighted as a hinge residue using the DC technique (Fig. 7), indicating the importance of incorporating chemical interactions into the AAN construction.

Residue H178 binds the phosphate moiety of glyceraldehyde 6-phosphate in the enzyme glyceraldehyde 6-phosphate dehydrogenase (G6PD). Like G157, this residue also forms a hinge in the network: of 485 residues H178 is ranked 9th for closeness centrality (0.14) and 464th for degree centrality (0.004). By making few interactions, it is able to leave chemical groups free to bind the phosphate group via hydrophilic interactions. The low degree also results in its high identified flexibility (−1.1 *kcal*/*mol*) using CNA. Indeed, via site-directed mutagenesis H178 is found to contribute 1.4 *kcal*/*mol* net to the binding of G6P and is found conserved in all 27 G6PDs sequenced up to 1998^37^.

For the majority of residues, the strength of an interaction does not appear to strongly influence the centrality (Fig. S2). In addition, the high closeness centrality bin also displays a greater spread, suggesting that residues that make fewer interactions can still have a high closeness centrality. The above hinge residues exemplify the importance of considering more complex measures of centrality and how the combination of different centrality measures can help determine the role of a residue.

Hinge residues in proteins are found to behave as centres for global motion, displaying dynamic stability and strong conservation^38^,^39^. We have shown that, for proteins in the data set, residues with lower degree generally have higher flexibility (Fig. S3) and that among these residues those with high closeness are more strongly conserved than hub residues. The overlap between residues that display strong evolutionary dynamics and large structural dynamics suggests that conservation exists in the protein sequence to maintain motion^40^. The RGN hinge residues share several characteristics with protein hinge residues, which suggests that they can be used to identify centres for hinge motion and investigate evolutionary dynamics.

### Allosteric Analysis of GPCR signalling

The family of G-protein coupled receptors (GPCRs) includes proteins that are involved in signal transmission across the lipid membrane. GPCRs contain a transmembrane core of seven *α*-helices (H1-H7) that facilitate signal transduction. One such GPCR is rhodopsin, which is found in the rod cells of the retina where incident photons trigger a cascade of events that ultimately result in an electrical signal being passed from the eye to the brain. In order to absorb light, the protein binds 11-*cis*-retinal at the interface between H5, H6, and H7 via a covalent bond to Lys296 on H7. Isomerisation to *all*-*trans*-retinal occurs when an incident photon is absorbed by retinal. The *cis*-to-*trans* conversion undergone by this small compound is amplified by nearby residues, causing structural changes at distant, allosteric residues that stabilise the active conformation of the protein^41^.

EVT is a random walk based measure of node importance during signal propagation. This holistic measure shares important features with allostery, including multiple pathway exploration and communication with distant sites. EVT analysis of rhodopsin has been previously carried out using constraints identified from molecular dynamics simulations^20^, where correlations in atomic motion were used to build the edges of the network. By initiating a signal at the retinal molecule, a series of allosteric regulatory sites were identified using this dynamically-constructed network. In order to investigate whether the same insight into allosteric communication in rhodopsin may be obtained using the computationally cheaper AAN construction technique developed in this paper, we have constructed the wRGN of rhodopsin and performed an EVT analysis of the resulting network. Finally, we have also compared the network properties of the DC approach, which is computationally inexpensive but may be too simplistic to capture the chemistry of the interaction network in rhodopsin.

**Figure 8:**
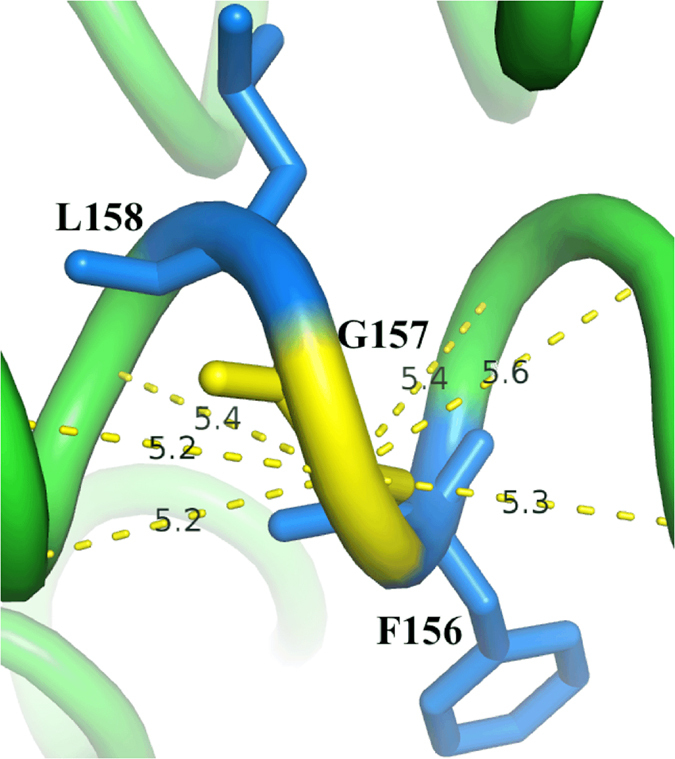
The ionic lock, which stabilises the inactive conformation and is broken in response to photon absorption, was found to display below average scaled-EVT values in DC network (**A**) and significantly high scaled-EVT values in the wRGN (**B**). Residues with high scaled-EVT values are coloured red and have greater thickness.

In general, the scaled-EVT values of the dynamical and RGN networks displayed sharp inhomogeneity, which peak at residues that regulate allosteric responses to photon absorption. Often, such residues were not highlighted in the DC network (Table 2). For example, spin label studies^42^ have previously revealed structural changes at F313 in response to photon absorption. R135 is a key allosteric residue, as it forms part of the most highly conserved motif in GPCRs, known as the DRY motif^43^. This residue forms the strongest hydrophilic interaction (*E* = −9.93 *kcal*/*mol*) that is observed in the network with neighbouring residue E134, which also exhibits a high scaled-EVT value. This pair of residues can be found in topologically identical locations in most GPCRs and is predicted to stimulate the release of GDP as a result of photo-activation^44^. The “ionic lock”, which describes the interaction between residues R135 and E247, stabilises the inactive conformation and is broken in response to photon absorption, allowing a shift to the active conformation^45^. We find that R135 displays the highest scaled-EVT value in the 326 node wRGN, but is not highlighted in the DC network (Fig. 8). Due to the low resolution of the rhodopsin crystal structure (3.4 *Å*), we find an abnormally short distance between the interacting groups of R135 and E247. The energy function used in FIRST thus penalises the interaction, assigning a positive energy to the salt bridge^10^. Under the H_*cut*_ imposed to construct the RGN, this interaction is not included in the network. If we add a modest salt bridge of −5.0 *kcal*/*mol* between the network nodes and re-run the EVT analysis, we find little perturbation to the previously calculated values with an average difference in the observed scaled EVT values of 0.13. This change is largely due to an increase in the EVT of R135 from 3.79 to 4.71. Noteably, the introduction of this interaction results in a high scaled EVT value for E247 (2.78) that is not identified in the DC approach (−0.89). This analysis highlights the importance of using high resolution crystal structures when constructing the RGN.

**Figure 9:**
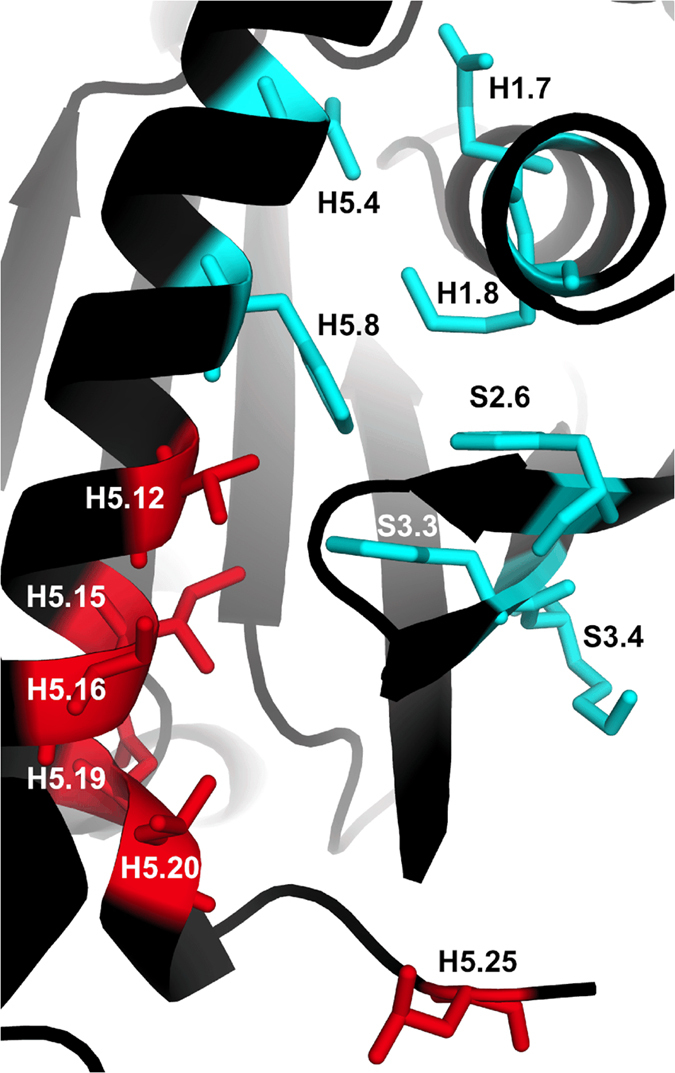
CGN positions on the inactive G*α* structure (pdb:1AS3). Residue positions highlighted using the average EVT analysis (cyan) form part of an allosteric wire involved in GDP release. Residues found in red form contacts with the GEF.

Membrane embedded GPCRs, such as rhodopsin, initiate downstream signalling events using G-proteins. In particular, GPCRs can act as guanine nucleotide exchange factors (GEFs), which bind to the *α* subunit of G-proteins (G*α*) and stimulate the release of GDP. The empty pocket is then free to bind GTP, which acts as a molecular switch for the active conformation of G*α*. The process of GDP release employs allosteric communication as the GEF does not interact with GDP directly. A common G*α* numbering (CGN) system (http://www.mrc-lmb.cam.ac.uk/CGN) has been identified for the 390 residue positions found in the G*α* protein family and was herein used to compare the EVT signals of homologous structures. To study the signaling pathway, we have taken average scaled EVT values of 10 inactive G*α* structures, and simulated perturbations at the sites in *α*-helix 5 (H5) that undergo contact rewiring in the GEF-bound state. These positions, namely G.H5.12,15,16,19,20,25, form between 3 and 5 contacts at the G*α*-GEF interface (Fig. 9, red). We calculate an inactive G*α* EVT signal by taking the average scaled EVT across the structures. We find that the CGN positions displaying the highest average scaled EVT for the 6 initiation sites overlap strongly with the conserved allosteric “wire” identified previously^46^ (Fig. S4).

The G*α*-GEF interface ultimately gives rise to helical domain opening that allows the dissociation of GDP. The highest scaled EVT is found for positions G.H5.4 (3.46) and G.H5.8 (3.35) (Fig. 9, cyan), which correspond to the 4^*th*^ and 8^*th*^ positions of *α*-helix 5 in the G domain. G.H5.4 has been highlighted in previous experiments as being important for G*α*-GDP stability. Indeed, this site forms the most contacts in the inactive state, the majority of which are interrupted when binding to GEF^46^. Mutation to G.H5.4 also gives rise to the greatest instability of the G*α*-GDP state of any residues in H5. Ala and Cys mutations to G.H5.8 accelerate GDP exchange, as it forms conserved contacts with H1. Indeed, several universally conserved contacts maintain the link between H5 and H1 in the absence of the GEF, including G.S2.6 (EVT = 2.51) with G.S3.3 (1.84) and G.H1.8 (2.56) with G.H5.8. In the above residues, receptor binding causes a reorganisation of the contacts, ultimately disassociating H1 from H5 that facilitates GDP release. In addition to G.H1.8, we do find a high scaled EVT value for G.H1.7 (2.41), whose mutation causes the greatest decrease in stability in the G*α*-GEF complex out of all mutations to H1, as well as a modest decrease in G*α*-GDP stability^46^.

In summary, the wRGN is able to identify several allosteric residues as having high-scaled EVT values. This suggests that the computationally cheap, static AAN construction technique presented here can successfully identify allosteric residues in proteins involved in GPCR signalling, which had previously only been accomplished using dynamical techniques or with the aid of sequence alignments.

## Conclusions and Future Work

In this work we have presented a novel method for pseudo-dynamical construction of a weighted and an unweighted AAN using the geometric tool, FIRST. The information extracted from the physico-chemical environment allows energies to be assigned to the hydrogen bonds and salt bridges, weights to be imposed, and an energy cutoff to be established. We have calculated the degree, betweenness, and closeness centrality of residues belonging to 795 proteins. These parameters of the network were shown to display a strong, negative correlation with the evolutionary rates (Table 1). This indicates that mutations to residues that are less central are more likely to become fixed, and that for more central residues, mutations are less tolerable.

We hope to use the RGN to identify generalisable structural and functional signatures. Indeed, structural signatures can be investigated by searching for homologous network structures in protein families and observing how those networks are affected by different (protein) environments. Functional signatures will be identified by looking for the network of residues that are preserved in proteins with homologous functions. As low-frequency motion is conserved in protein families^47^, studying the sparse network of residues in the RGN that give rise to these functionally relevant motions could provide an additional route to study protein evolution. Furthermore, RGN analysis has hinted that although the three centrality measures employed successfully identify central residues, by combining and correlating different measures of centrality we can gain additional insight. This is exemplified by hinge residues (Figs 5 and 6) that connect functional regions and are likely (based on previous experimental studies) to play an important role in protein dynamics. Importantly, such hinge residues are not be highlighted using the DC-construction technique. Allosteric residues also tend to lie hidden in the DC network and readily emerge in the wRGN, suggesting that a deeper understanding of the non-covalent interactions is required to properly identify such residues. Although dynamical techniques can be used employed toward this end, the RGN provides a low-cost computational tool that can be readily applied to large protein data sets in search of network-function signatures.

## Additional Information

**How to cite this article**: Fokas, A. S. *et al*. Residue Geometry Networks: A Rigidity-BasedApproach to the Amino Acid Network and Evolutionary Rate Analysis. *Sci. Rep.* **6**, 33213; doi: 10.1038/srep33213 (2016).

## Supplementary Material

Supplementary Information

## Figures and Tables

**Table 1:** Correlation coefficient for the best performing value of H_*cut*_ for the unRGNs (rows 1–3) and wRGN for betweenness centrality (row 4).

Centrality Measure	H_*cut*_ (*kcal*/*mol*)	Correlation Coefficient
Degree	−2.0	−0.994
Betweenness	−2.5	−0.995
Closeness	−3.0	−0.997
Weighted Betweenness	−2.5	−0.997

**Table 2:** Scaled-EVT values for RGN, Dynamical AAN^20^, and DC AAN.

Residue	RGN	Dynamical	DC
W126	2.94	2.23	1.24
Y178	1.32	2.71	0.41
F103	0.58	2.25	−0.17
D83	−0.09	1.35	0.95
N55	−0.47	1.32	0.64
Y306	0.27	1.39	−0.82
F313	1.62	1.32	−0.82
V139	1.32	0.44	0.40
R135	3.79	1.07	0.55
E122	2.82		1.91
E134	1.35		0.50

Standardised values have been displayed to allow accurate comparison. Residues with high-scaled EVT values regulate allosteric change in rhodopsin and are often not identified using the DC network. We have left blank the dynamical column in cases where the residues have not been discussed in their study.

## References

[b1] Yan W. . The construction of an amino acid network for understanding protein structure and function. Amino acids 46, 1419–1439 (2014). 24623120 10.1007/s00726-014-1710-6

[b2] Greene L. H. & Higman V. A. Uncovering network systems within protein structures. J. Mol. Biol. 334, 781–791 (2003). 14636602 10.1016/j.jmb.2003.08.061

[b3] Zhou J., Yan W., Hu G. & Shen B. Amino acid network for the discrimination of native protein structures from decoys. Curr. Protein Pept. Sci. 15, 522–528 (2014). 25059328 10.2174/1389203715666140724084709

[b4] Goodey N. & Benkovic S. Allosteric regulation and catalysis emerge via a common route. Nat. Chem. Biol. 4, 474–482 (2008). 18641628 10.1038/nchembio.98

[b5] Boehr D. D., Nussinov R. & Wright P. E. The role of dynamic conformational ensembles in biomolecular recognition. Nat. Chem. Biol. 5, 789–796 (2009). 19841628 10.1038/nchembio.232 PMC2916928

[b6] Yang L.-W. & Bahar I. Coupling between catalytic site and collective dynamics: A requirement for mechanochemical activity of enzymes. Struct. 13, 893–904 (2005). 10.1016/j.str.2005.03.015 PMC1489920 15939021

[b7] Sistla R. K., Brinda K. V. & Vishveshwara S. Identification of domains and domain interface residues in multidomain proteins from graph spectral method. Proteins 59, 616–626 (2005). 15789418 10.1002/prot.20444

[b8] Bhattacharyya M., Bhat C. R. & Vishveshwara S. An automated approach to network features of protein structure ensembles. Protein Sci. 22, 1399–1416 (2013). 23934896 10.1002/pro.2333 PMC3795498

[b9] Wells S., Menor S., Hespenheide B. & Thorpe M. Constrained geometric simulation of diffusive motion in proteins. Phys. Bio. 2 (2005). 10.1088/1478-3975/2/4/S07 16280618

[b10] Wells S. A. Geometric simulation of flexible motion in proteins. In Livesay D. R. (ed.) Protein Dynamics Vol. II., Methods in Molecular Biology. 173–192 (Humana Press, New York, 2013). 10.1007/978-1-62703-658-0_10 24061922

[b11] Cheng T. M. K., Lu Y.-E., Vendruscolo M., Lio’ P. & Blundell T. L. Prediction by graph theoretic measures of structural effects in proteins arising from non-synonymous single nucleotide polymorphisms. PLoS Comput Biol 4, e1000135 (2008). 18654622 10.1371/journal.pcbi.1000135 PMC2447880

[b12] Dahiyat B. I., Gordon D. B. & Mayo S. L. Automated design of the surface positions of protein helices. Protein Sci 6, 1333–1337 (1997). 9194194 10.1002/pro.5560060622 PMC2143725

[b13] Wells S., Jimenez-Roldan J. E. & Romer R. Comparative analysis of rigidity across protein families. Phys. Bio. 6 (2009). 10.1088/1478-3975/6/4/046005 19773604

[b14] Jolley C. C., Wells S. A., Hespenheide B. M., Thorpe M. F. & Fromme P. Docking of photosystem I subunit C using a constrained geometric simulation. J. Am. Chem. Soc. 128, 8803–8812 (2006). 16819873 10.1021/ja0587749

[b15] Kozuska J. L. . Impact of intracellular domain flexibility upon properties of activated human 5-HT3 receptors. Br. J. Pharmacol. 171, 1617–1628 (2014). 24283776 10.1111/bph.12536 PMC3966743

[b16] Fulle S., Christ N. A., Kestner E. & Gohlke H. HIV-1 TAR RNA spontaneously undergoes relevant apo-to-holo conformational transitions in molecular dynamics and constrained geometrical simulations. J. Chem. Inf. Model. 50, 1489–1501 (2010). 20726603 10.1021/ci100101w

[b17] Belfield W. J., Cole D. J., Martin I. L., Payne M. C. & Chau P. L. Constrained geometric simulation of the nicotinic acetylcholine receptor. J. Mol. Graphics Model. 52, 1–10 (2014). 10.1016/j.jmgm.2014.05.001 24955489

[b18] Fokas A. S., Cole D. J. & Chin A. W. Constrained geometric dynamics of the Fenna–Matthews–Olson complex: the role of correlated motion in reducing uncertainty in excitation energy transfer. Photosynth. Res. 122, 275–292 (2014). 25034014 10.1007/s11120-014-0027-3

[b19] Franzosa E. A. & Xia Y. Structural determinants of protein evolution are context-sensitive at the residue level. Mol. Biol. and Evol. 26, 2387–2395 (2009). 19597162 10.1093/molbev/msp146

[b20] Park K. & Kim D. Modeling allosteric signal propagation using protein structure networks. BMC Bioinformatics 12, S23 (2011). 21342553 10.1186/1471-2105-12-S1-S23 PMC3044278

[b21] Yang Z. PAML: A program package for phylogenetic analysis by maximum likelihood. Comput. Appl. Biosci. 13, 555–6 (1997). 9367129 10.1093/bioinformatics/13.5.555

[b22] Nielsen R. Statistical Methods in Molecular Evolution. Statistics for Biology and Health (Springer, New York, 2006).

[b23] Beazley D. Python Essential Reference (Addison-Wesley, 2009).

[b24] Hagberg A. A., Schult D. A. & Swart P. J. Exploring network structure, dynamics, and function using NetworkX. In *Proceedings of the 7th Python in Science Conference (SciPy2008),* 11–15 (Pasadena, CA USA, 2008).

[b25] Davis I. W. . Molprobity: all-atom contacts and structure validation for proteins and nucleic acids. Nucleic Acids Res 35, W375–W383 (2007). 17452350 10.1093/nar/gkm216 PMC1933162

[b26] Grant B. J., Rodrigues A. P. C., ElSawy K. M., McCammon J. A. & Caves L. S. D. BIO3D: an R package for the comparative analysis of protein structures. Bioinform. 22, 2695–2696 (2006). 10.1093/bioinformatics/btl461 16940322

[b27] Kemeny J. & Snell J. Finite Markov Chains. Undergraduate Texts in Mathematics (Springer, New York, 1983).

[b28] del Sol A., Fujihashi H., Amoros D. & Nussinov R. Residue centrality, functionally important residues, and active site shape: Analysis of enzyme and non-enzyme families. Protein Science 15, 2120–2128 (2006). 16882992 10.1110/ps.062249106 PMC2242611

[b29] Pfleger C., Rathi P. C., Klein D. L., Radestock S. & Gohlke H. Constraint network analysis (CNA): A python software package for efficiently linking biomacromolecular structure, flexibility, (thermo-)stability, and function. J Chem Inf Model 53, 1007–1015 (2013). 23517329 10.1021/ci400044m

[b30] Krüger D. M., Rathi P. C., Pfleger C. & Gohlke H. Cna web server: rigidity theory-based thermal unfolding simulations of proteins for linking structure, (thermo-)stability, and function. Nucleic Acids Res 41, W340–W348 (2013). 23609541 10.1093/nar/gkt292 PMC3692064

[b31] Artz J. D. . Molecular characterization of a novel geranylgeranyl pyrophosphate synthase from plasmodium parasites. J. Biol. Chem. 286, 3315–3322 (2011). 21084289 10.1074/jbc.M109.027235 PMC3030337

[b32] Kavanagh K. L., Dunford J. E., Bunkoczi G., Russell R. G. G. & Oppermann U. The crystal structure of human geranylgeranyl pyrophosphate synthase reveals a novel hexameric arrangement and inhibitory product binding. J. Biol. Chem. 281, 22004–22012 (2006). 16698791 10.1074/jbc.M602603200

[b33] Olah G. A., Mitchell R. D., Sosnick T. R., Walsh D. A. & Trewhella J. Solution structure of the cAMP-dependent protein kinase catalytic subunit and its contraction upon binding the protein kinase inhibitor peptide. Biochemistry 32, 3649–3657 (1993). 8385485 10.1021/bi00065a018

[b34] Zheng J., Knighton D., Xuong N., Taylor S., Sowadski J. & Teneyck L. Crystal structures of the myristylated catalytic subunit of cAMP-dependent protein kinase reveal open and closed conformations. Protein Sci. 2, 1559–1573 (1993). 8251932 10.1002/pro.5560021003 PMC2142252

[b35] Teplyakov A. . Crystal structure of bacteriophage T4 deoxynucleotide kinase with its substrates dGMP and ATP. EMBO JD 15, 3487–3497 (1996). PMC451945 8670851

[b36] Narayana N., Cox S., Xuong N., Ten Eyck L. & Taylor S. A binary complex of the catalytic subunit of cAMP-dependent protein kinase and adenosine further defines conformational flexibility. Structure 5, 921–935 (1997). 9261084 10.1016/s0969-2126(97)00246-3

[b37] Cosgrove M. S., Naylor C., Paludan S., Adams M. J. & Levy H. R. On the mechanism of the reaction catalyzed by glucose 6-phosphate dehydrogenase. Biochem. 37, 2759–2767 (1998). 9485426 10.1021/bi972069y

[b38] Liu Y. & Bahar I. Sequence evolution correlates with structural dynamics. Molecular Biology and Evolution 29, 2253–2263 (2012). 22427707 10.1093/molbev/mss097 PMC3424413

[b39] Nevin Gerek Z., Kumar S. & Banu Ozkan S. Structural dynamics flexibility informs function and evolution at a proteome scale. Evolutionary Applications 6, 423–433 (2013). 23745135 10.1111/eva.12052 PMC3673471

[b40] Marsh J. A. & Teichmann S. A. Parallel dynamics and evolution: Protein conformational fluctuations and assembly reflect evolutionary changes in sequence and structure. BioEssays 36, 209–218 (2014). 24272815 10.1002/bies.201300134

[b41] Kessel A. & Ben-Tal N. Introduction to Proteins: Structure, Function, and Motion. Chapman & Hall/CRC Mathematical and Computational Biology (CRC Press, 2010).

[b42] Altenbach C., Cai K., Khorana H. G. & Hubbell W. L. Structural features and light-dependent changes in the sequence 306–322 extending from helix VII to the palmitoylation sites in rhodopsin:? a site-directed spin-labeling study. Biochemistry 38, 7931–7937 (1999). 10387035 10.1021/bi9900121

[b43] Sakmar T. P., Menon S. T., Marin E. P. & Awad E. S. Rhodopsin: Insights from recent structural studies. Annu. Rev. Biophys. Biomol. Struct. 31, 443–484 (2002). 11988478 10.1146/annurev.biophys.31.082901.134348

[b44] Acharya S. & Karnik S. S. Modulation of GDP release from transducin by the conserved Glu134-Arg135 sequence in rhodopsin. J Bio Chem 271, 25406–25411 (1996). 8810308 10.1074/jbc.271.41.25406

[b45] Ahuja S. . Helix movement is coupled to displacement of the second extracellular loop in rhodopsin activation. Nat Struct Mol Biol 16, 168–75 (2009). 19182802 10.1038/nsmb.1549 PMC2705779

[b46] Flock T. . Universal allosteric mechanism for ga activation by gpcrs. Nature 524, 173–179 (2015). 26147082 10.1038/nature14663 PMC4866443

[b47] Echave J. Why are the low-energy protein normal modes evolutionarily conserved? Pure Appl. Chem. 84, 1931–1937 (2012).

