# Corrigendum: Residue Geometry Networks: A Rigidity-Based Approach to the Amino Acid Network and Evolutionary Rate Analysis

**DOI:** 10.1038/srep42654

**Published:** 2017-02-27

**Authors:** Alexander S. Fokas, Daniel J. Cole, Sebastian E. Ahnert, Alex W. Chin

Scientific Reports 6: Article number: 33213; 10.1038/srep33213 published online: 09 14 2016; updated: 02 27 2017.

In this Article, the legend of Figure 6 is incorrect:

A portion of the RGN of GGPPS is shown, displaying interactions made by coloured nodes. It can be seen that G157 interacts with only two residues (low degree). However, these residues form extensive interactions with the environment, and therefore give rise to the high closeness of G157. Critically, the blue nodes are known to play important roles in ligand binding.

should read:

The ionic lock, which stabilises the inactive conformation and is broken in response to photon absorption, was found to display below average scaled-EVT values in DC network (A) and significantly high scaled-EVT values in the wRGN (B). Residues with high scaled-EVT values are coloured red and have greater thickness.

In addition, the legend of Figure 8 is incorrect:

The ionic lock, which stabilises the inactive conformation and is broken in response to photon absorption, was found to display below average scaled-EVT values in DC network (A) and significantly high scaled-EVT values in the wRGN (B). Residues with high scaled-EVT values are coloured red and have greater thickness.

should read:

A portion of the RGN of GGPPS is shown, displaying interactions made by coloured nodes. It can be seen that G157 interacts with only two residues (low degree). However, these residues form extensive interactions with the environment, and therefore give rise to the high closeness of G157. Critically, the blue nodes are known to play important roles in ligand binding.

