# Supplementary material for: Residue Geometry Networks: A Rigidity-Based Approach to the Amino Acid Network and Evolutionary Rate Analysis
Source: Sci Rep. 2016 Sep 14;6:33213. doi: 10.1038/srep33213 (PMC5021933; doi:10.1038/srep33213)
Supplement: Supplementary Information [file srep33213-s1.pdf]

Supporting Information for : “Residue Geometry Networks: A Rigidity-Based Approach to the Amino Acid Network and Evolutionary Rate Analysis”

Alexander S. Fokas[1,*], Daniel J. Cole[1], Sebastian E. Ahnert[1], and Alex W. Chin[1]

[1]Theory of Condensed Matter Group, Cavendish Laboratory, 19 JJ Thomson Avenue, CB3 0HE, Cambridge, U.K.
[*]asf40@cam.ac.uk

Author Contributions

The authors declare no competing financial interests. ASF carried out data collection and analysis and wrote the main script. DJC, AWC, SEA supervised the work. All authors reviewed the manuscript.

Supplementary Figures

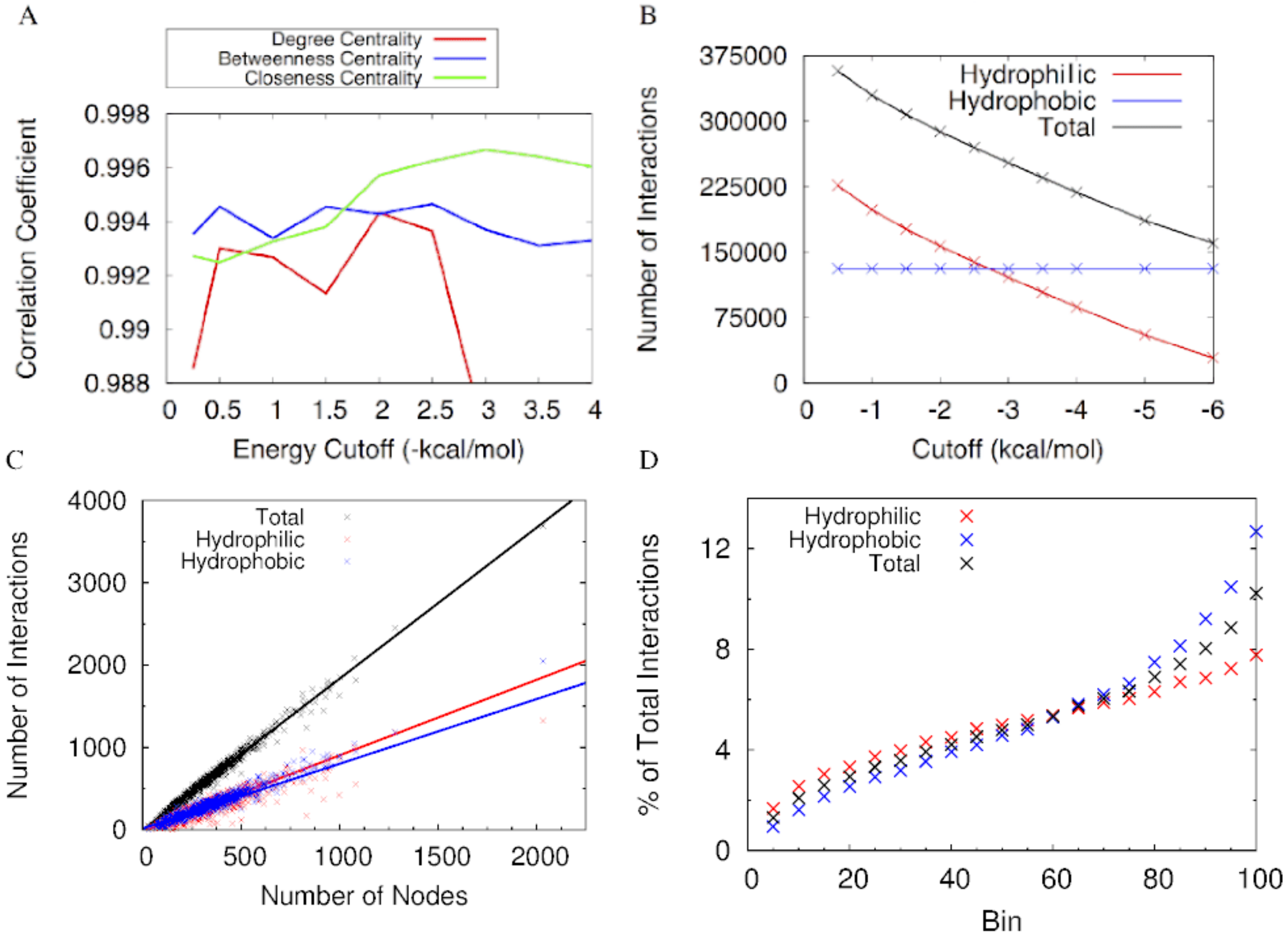

Figure S1. (A) The effect of varying the H_{cut} parameter on correlation coefficient (as measured in Fig. 2 in the main text) for the unRGNs. (B) The number of hydrophilic, hydrophobic, and total interactions as a function of cutoff. Although the results of the analysis using a H_{cut} of -6.0 and -8.0 $kcal/mol$ were less correlated with evolutionary rate than higher H_{cut} values, a correlation coefficient of > 0.99 was still observed. The robustness of the analysis to H_{cut} stems from the treatment of hydrophobic interactions. Namely, as the hydrophobic interaction energies are not explicitly calculated, they are not removed when lowering the value of H_{cut} and the total number remains constant. These interactions can therefore still identify high centrality residues at extremely low values of H_{cut} where very few hydrophilic interactions are involved, particularly in the centre of the protein where hydrophobic interactions are mostly concentrated. (C) For each protein the number of nodes has been plotted against the number of hydrophilic, hydrophobic, and total non-covalent interactions. (D) The percentage of the total number of hydrophilic, hydrophobic, and total interactions made by all residues in the data set are displayed for each bin. The closeness centrality bins for the unRGN were employed for B-D at a H_{cut} of -3.0 $kcal/mol$.

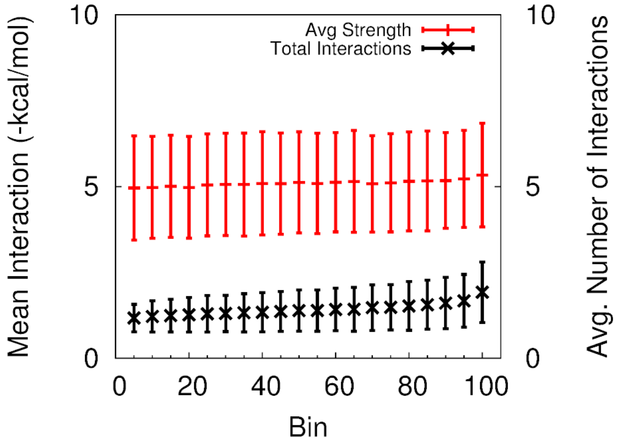

Figure S2. The average strength of the hydrophilic interactions made by residues in each bin is displayed using red crosses, with the error bars representing the standard deviation. The black crosses (right axis) show the average number of hydrophilic interactions made by the residues in each bin. Note, for each residue in the bin the strength of all hydrophilic interactions formed are summed to give a single value for each residue.

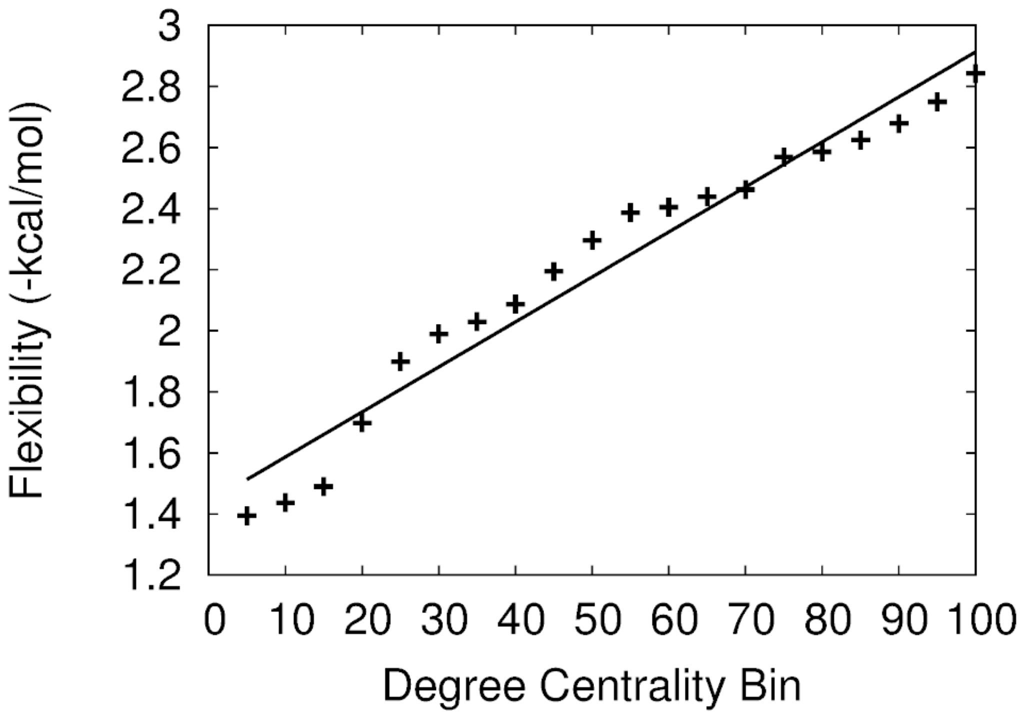

Figure S3. Correlation between average bin rigidity index and degree centrality bin. The results show a strong, negative correlation ($r = -0.98$) between average rigidity index and degree centrality bin. Therefore, residues with a lower degree are more flexible.

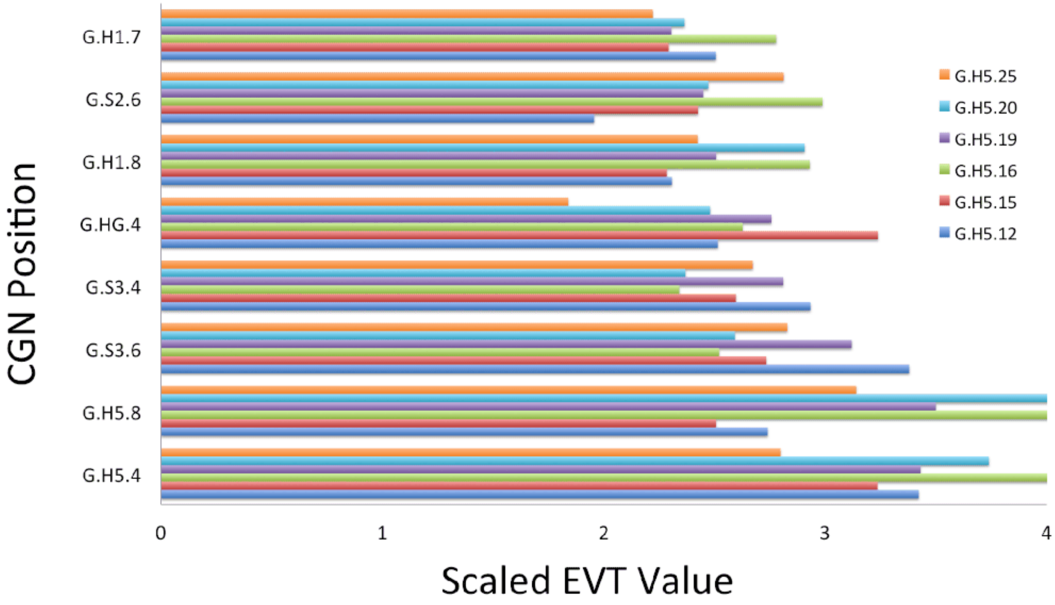

Figure S4. We have taken the average scaled EVT at each CGN position using 10 inactive pdb structures (3AH8, 1ZCB, 1AS3, 3UMS, 1TAG, 3UMR,3FFB, 1GG2, 1GP2, 1GOT). Then, by considering signal absorption at positions that interact with the GEF, namely G.H.[12,15,16,19,20,25], we have taken the average scaled EVT measured with respect to these residues at the CGN positions to investigate where the signalling from these residues is most sensitive. Above are the residues with the highest average, with respect to the GEF interacting residues, scaled EVT values, within which several residues overlap with a conserved allosteric wire identified in Gα proteins.
